# Being an Infant in a Pandemic: Influences of the COVID-19 Pandemic on Infants, Toddlers and Their Mothers in a Clinical Population

**DOI:** 10.3390/children10121885

**Published:** 2023-12-01

**Authors:** Mona Katharina Sprengeler, Janna Mattheß, Mirijam-Griseldis Galeris, Melanie Eckert, Gabriele Koch, Thomas Reinhold, Anne Berghöfer, Julia Fricke, Stephanie Roll, Thomas Keil, Christiane Ludwig-Körner, Lars Kuchinke, Kai von Klitzing, Lars Otto White, Franziska Schlensog-Schuster

**Affiliations:** 1 Department of Child and Adolescent Psychiatry, Psychotherapy and Psychosomatics, University of Leipzig, 04103 Leipzig, Germany; mona.sprengeler@medizin.uni-leipzig.de (M.K.S.); mirijam-griseldis.galeris@medizin.uni-leipzig.de (M.-G.G.); kai.klitzing@medizin.uni-leipzig.de (K.v.K.); larsotto.white@medizin.uni-leipzig.de (L.O.W.); franziska.schlensogschuster@upd.ch (F.S.-S.); 2 International Psychoanalytic University, 10555 Berlin, Germany; melanie.eckert@ipu-berlin.de (M.E.); gabriele.koch@ipu-berlin.de (G.K.); christiane.ludwig-koerner@ipu-berlin.de (C.L.-K.); lars.kuchinke@ipu-berlin.de (L.K.); 3 Institute of Social Medicine, Epidemiology and Health Economics, Charité—Universitätsmedizin Berlin, 10117 Berlin, Germany; thomas.reinhold@charite.de (T.R.); julia.fricke@charite.de (J.F.); stephanie.roll@charite.de (S.R.); thomas.keil@charite.de (T.K.); 4 Institute of Clinical Epidemiology and Biometry, University of Wuerzburg, 97080 Wuerzburg, Germany; 5 State Institute of Health, Bavarian Health and Food Safety Authority, 97688 Bad Kissingen, Germany; 6 University Hospital of Child and Adolescent Psychiatry and Psychotherapy, University of Bern, 3000 Bern, Switzerland

**Keywords:** COVID-19 pandemic, maternal mental health, regulatory disorders, mother-child-interaction, emotional availability, infants and toddlers

## Abstract

The COVID-19 pandemic and the ongoing lockdowns might have had a strong impact on mental health of mothers and their infants/toddlers. For example, families had to deal with health issues and social isolation, which might have affected mental health and parent-child interactions. The aim of this study is to evaluate differences in (1) infantile regulatory disorders, (2) maternal mental health, (3) the impact of maternal mental health on infantile regulatory disorders, and (4) alterations in the mother-child interaction for participants recruited before versus after the onset of the first German lockdown. For this reason, mother-child dyads have been divided into two groups and were compared by analyzing clinical interviews on psychopathology of mother and child (M.I.N.I. & DC:05) and mother-child-interactions (Emotional Availability Scales). Results showed that (1) differences in infantile sleeping disorders emerged (*phi* = 0.243; *p* = 0.016) compared to the pre-lockdown group, while (2) the occurrence of maternal panic and anxiety increased in the post-lockdown group (*phi* = 0.229; *p* = 0.022). Moreover, there was (3) an association for maternal panic and child’s sleep disorder, and (4) specific associations with maternal non-hostility in the mother-child-interaction. In conclusion, the present study highlights the differences of maternal mental health occurrences and infants’ regulatory problems, as well as the possible effects of the COVID-19 pandemic for infants. In the pre-lockdown group, maternal non-hostility might have acted as a promotive factor against regulatory disorders, while this mechanism was mitigated in the post-lockdown group.

## 1. Introduction

The COVID-19 pandemic affected many people and their day-to-day life, with a strong impact on families around the globe [1]. Based on research, it can be assumed that it had influences on parental, infant and toddlers’ mental health. On 16 March 2020, Germany’s first lockdown started, shortly after the first infection was detected in the country. Since that day, Germany was in lockdown off and on again until the 20 March 2022 [2]. Various social-distancing policies were enacted in Germany, like the implementation of nationwide stay-at-home policies (“lockdown-weeks”) with the closure of nurseries, kindergartens, and schools (except for children of parents with systemically relevant professions, e.g., doctors), contact restrictions, wearing masks outside at home, and closing shops. After an infection, people were quarantined (and isolated) for at least 10 days.

During the COVID-19 pandemic, depression and anxiety levels rose even within people without any previous history of psychological disorders [3]. The pandemic came along with many stressors, e.g., the anxiety of a severe disease, trauma caused by (sudden) loss, coping with symptoms, and separation [4]. These contributing factors have a known impact on psychological health. Saccone et al. [5] and Moyer et al. [6] verified significantly increased anxiety levels in pregnant women very early in the pandemic. King et al. [7] detected in a large sample of 725 women that pregnancy during the pandemic raised twice the risk of developing depressive symptoms compared to women being pregnant before the pandemic. Even postpartum, mothers reported higher levels of depressive symptoms during the pandemic. The combination of daily care-taking, home day care and home schooling while coordinating work demands, their own anxiety, and concerns about their children’s development [8], as well as social isolation outside the household caused an immense stressful family reality, apart from physical health problems. From the child’s perspective, the pandemic caused social isolation, caused by the strict closure of child day care centers (except for children of parents with systemically relevant professions, e.g., doctors) and different (negative) developmental challenges (like speech development when wearing a mask, motor, fine, and social development when isolated from peers) [9,10].

While it has already been shown that before the COVID-19 pandemic, parental mental health problems (like depression, panic and anxiety, obsessive-compulsive disorder) contribute to a child’s risk of developing a psychopathology [11], the social distancing policy seemed to multiply the effects of distress and psychological symptoms [12]. Studies demonstrated that maternal distress caused by the COVID-19 pandemic already impacts infant’s regulation capacity in the first three months of life and is generally connected with early regulatory disorders [13]. Georg et al. [14] summed up in 2021 when writing: “Children are inherently vulnerable because they depend on adults to have their most basic needs met. When those adults lack the wherewithal to cope with the immediate, urgent, and multiple adaptive demands a pandemic places on families and when support systems do not exist, falter, or cease, it can result in unmitigated disaster for the very young” [14] (p. 760). Before the pandemic, already about 20% of infants and toddlers were affected by at least one regulatory disorder (e.g., anxiety, excessive crying, eating or sleep disorders) [11,15]. During the COVID-19 pandemic, mothers described an increase in crying and sleeping disorders in their infants seven months postpartum [16]. To this day, it is unknown to what extent the pandemic and ongoing lockdowns influenced maternal psychopathology and the child’s regulatory disorders in the first three years of life, and if the number of disorders increased during the lockdowns.

Furthermore, infants and toddlers in the pandemic spent most of their time in social isolation, so that they were singularly focused on their parent/sibling-interactions during the phase of lockdowns. Lordi and Tan [17] reported that families had problems supporting their children during the phase of lockdowns with still visible alterations of early parent-child interaction. One of the key elements of early parent-child interaction is the parental emotional availability (EA). EA is described as integral for healthy development and infantile psychological well-being, as well as social- and cognitive development [18,19,20,21,22]. In general, higher maternal EA is connected to fewer behavioral symptoms in infancy and childhood [23,24] and might act as a promotive factor interrupting the trajectories of risks [25]. Promotive factors in general are connected to a healthy development and overcoming obstacles [25,26], and could be leading towards higher resiliency against mental disorders in infancy and childhood as a final result [27]. It is well known that parental EA decreases under pressure—as shown during the pandemic situation worldwide [28]. In addition, studies pointed to the fact that parents showed more harsh parenting behaviors in parent-child relationships with children during the pandemic [29,30]. On the other hand, McRae et al. [31] showed that this effect might be buffered by partners’ support and co-parenting. Furthermore, a study extended these findings by underlining that mothers with younger children (age 3–6 years) developed more preservative child-rearing strategies during the pandemic compared to non-Covid situations [32]. In contrast, one study [33] found, nonetheless, no significant changes in maternal EA measured before and during the pandemic in infants and toddlers.

Taking these findings all together, there are no consistent results that the pandemic could have influenced the parental EA. Especially in the early phase of life (0–36 months), results are very rare. As it remains unclear whether maternal EA works as a promotive mechanism for infantile regulatory diagnoses in early childhood during the pandemic, the aim of this study is to examine if parental EA differed between pre- and post-lockdowns and if EA could be a contributing mechanism for infantile psychopathology during the COVID-19 pandemic and the ongoing lockdowns. In addition, and considering the literature on psychological well-being and child development, the assumption could be made, that even in the COVID-19 pandemic, high EA can be seen as a promotive factor leading to resiliency in children. With the aim to close this research gap, the present study is the first step examining not only the potential risks, but also protective and promotive factors in the mother-child-interaction.

The current study seeks to address the following questions:

1. Did maternal mental health symptoms differ in participants included before or after the onset of the first COVID-19 lockdown in Germany?
2. Did infantile regulatory disorders differ between participants included before or after the onset of the first COVID-19 lockdown?
3. Is there a difference between the influence of maternal mental health on the infantile regulatory disorders in participants included before and after the onset of the first lockdown of the COVID-19 pandemic?
4. Is there a difference in the maternal emotional availability for mother-child dyads included before or after the onset of the first lockdown?
5. In comparison to participants included before the onset of the first lockdown, can maternal EA be a promotive factor against infantile regulatory disorders?

## 2. Materials and Methods

### 2.1. Participants

Data of 140 mother-child dyads who have been enrolled in a randomized controlled trial (RCT) (see Section 2.2 and [34]) were part of this analysis. Of these, 130 participants completed baseline assessments. In total, 31 dyads were excluded due to missing data concerning the infant’s and toddler’s diagnosis acquired by the Diagnostic Classification of Mental Health and Developmental Disorders of Infancy and Early Childhood structured interview (DC:0–5; [35]). Therefore, a total of N = 99 mother-child dyads were included in data analysis. Of these, 40 dyads had been enrolled in the RCT before March 2020 (pre lockdown), 59 afterwards (post lockdown). All infants and toddlers were aged from 0–36 months and had been diagnosed with at least one regulatory disorder (e.g., excessive crying, sleeping, eating, mood disorder or separation anxiety; see Table S2 for detailed description).

For inclusion in the study, mothers had to speak German to a sufficient level and had to be in a psychological state which allowed them to participate in the study. Mother-child dyads who already participated in other clinical trials were excluded from study, as well as mothers or children receiving any other form of psychotherapy. Mothers dealing with acute substance abuse, suicidality, or psychosis could not participate in the trial. Data was conducted in in- and outpatient settings. Participants were either included before the first onset of a lockdown on 16 March 2020, or afterwards, the first mother-child dyads included in the post-lockdown group were recruited on 16 April 2020 (see Figure S1). For further information concerning the RCT design, see Sprengeler et al., 2021 [34].

### 2.2. Study Procedures

This study is part of the multicenter research project Evaluation of Parent-Infant Psychotherapy: epidemiologic cohort study and intervention studies (SKKIPPI; [34,36,37]) as part of a RCT, which evaluated the integrated psychological psychiatric care for families with children from 0–36 months. The overall project consists of a cohort study [36] and two randomized-controlled intervention trials (for further information see [34,37]), funded by the German Health Care Innovation Fund. Written informed consent was obtained from all participants and their legal guardians to participate in the RCT and use of their data in research related to the questions of the RCTs in anonymized form. The current analysis only contains baseline data (T0) of the RCT Children [34]. Baseline data have been measured during March 2019 and December 2021 for mother-child dyads included before (pre-lockdown; till 15 March 2020) and after (post-lockdown, from 16 March 2020; Figure S1) the onset of the first German lockdown (see Figure S1). The cut-off date is a simplification, and disorders that have an origin before the cut-off date may not be perfectly allocated.

### 2.3. Variables and Instruments

#### 2.3.1. Infant’s Regulatory Disorders

To assess the infants and toddlers’ regulatory disorders, trained research assistants conducted a self-constructed structured interview using the criteria of the DC: 0–5 Axis 1 with the mothers [34,35]. The structured interview covers 13 regulatory disorders of the DC: 0–5 and 4 main categories including sleeping disorders (subcategories: sleep onset disorder, night waking, partial arousal sleep, nightmares), eating disorders (subcategories: under eating disorder, overeating disorder and atypical eating disorder), mood disorders (subcategories: depression, dysregulated anger and aggression, other mood disorder), and other disorders (subcategories: sleeping disorder, eating disorders, mood disorders, excessive crying, other eating, sleep or crying disorder, and separation anxiety). For more information see Table S1. For the self-constructed DC: 0–5^TM^ no psychometric properties were available at time of data analysis.

#### 2.3.2. Maternal Mental Health

The Mini International Neuropsychiatric Interview was conducted to assess possible maternal clinical diagnoses (M.I.N.I.) [38]. The interview is an internationally recognized gold-standard survey instrument with good to very good psychometric properties compared to the CIDI (kappa coefficient between 0.36 and 0.82) and acceptable test-retest-reliability (kappa coefficient between 0.76 and 0.93) [38]. The M.I.N.I. was assessed by trained researchers and is allowed for administration by non-specialized interviewers, so no validation by a medical professional was obtained. The structured interview evaluates 20 psychiatric disorders by DSM IV and ICD-10, including affective disorders (subcategories: major depressive episode: current, major depressive episode: lifetime, major depressive episode with melancholia, dysthymia, suicidality, (hypo-)manic episode: current, (hypo-)manic episode: lifetime, generalized anxiety disorder), panic and anxiety disorders (subcategories: panic disorder: current, panic disorder: lifetime, panic disorder with few symptoms, panic disorder without agoraphobia, panic disorder with agoraphobia), phobic disorders (subcategories: agoraphobia without former panic disorder, social phobia), eating disorders (subcategories: anorexia nervosa, bulimia nervosa, anorexia nervosa: binge-eating), as well as psychotic disorders and antisocial personality disorder (see Table S2). A lifetime panic disorder can be diagnosed if a person had at least one panic attack, coupled with at least four specific panic symptoms (e.g., rise in heartbeat, shivering, hyperventilating) and the anxiety of another episode for at least one month.

#### 2.3.3. Mother-Child-Interaction

Mother-child interaction was evaluated with the Emotional Availability Scales (EAS) [19]. The EAS has good psychometric properties (ICC between 0.79 and 0.92 [39] and retest-reliability for sensitivity r_tt_ = 0.55 [40]) and assesses sensitivity, structuring, non-intrusiveness, non-hostility, child responsiveness, and child involvement. For each scale, a total score ranging from 7 to 29 is determined. Higher scores represent higher levels of emotional availability than lower scores. Considering this, the EAS were rated on 7-point scales (1 = non-optimal to 7 = optimal). The coders had been trained and accredited to use the EAS by Zeynep Biringen, one of the developers of the Emotional Availability Scales [19]. 15–20 min free-play interaction were videotaped and rated by independent and reliable coders who were blinded for further information of the mother-child dyads.

### 2.4. Statistical Analyses

For the present analyses, only cross-sectional comparisons of two groups (pre- and post-lockdown) have been conducted. In a first step, descriptive statistics were determined for the study sample (child’s and maternal age, child’s gender, maternal nationality, custody, maternal education level, relationship status, number of children per household, and financial situation) and compared between the pre- and post-lockdown group. Depending on the scale of the variables independent samples *t*-Tests or Chi-square statistics are reported. In the second step, the association for infantile regulatory disorders between the pre- and post-lockdown group were explored using phi coefficients for contingency tables with 2 binary variables. Phi coefficients are recommended when data has a bivariate binomial distribution. For this step, either the four main categories of infantile regulatory disorders were used to compute phi coefficients or a comorbidity score (cumulative score) of the four main categories. To evaluate comorbidity within and across the four main categories, a dichotomous variable was computed defined by being diagnosed with at least two disorders occurring simultaneously for the category of interest (=1), or not being diagnosed with two or more disorders (=0). Subsequently, when an association was evaluated at *p* ≤ 0.05, the subcategories of interest were exploratively examined in the same way.

To examine the association for maternal mental health problems between the pre- and post-lockdown group, phi coefficients were determined. For the analysis, maternal mental health problems were subsumed into four main categories: panic and anxiety, affective disorders, eating disorders and phobic disorders. Subsequently, when an association was evaluated at *p* ≤ 0.05, the subcategories of interest were exploratively examined in the same way. Fourthly, to examine the co-occurrences of infantile regulatory and maternal mental health problems, the associations of the four main categories of cumulative infantile regulatory disorders with the maternal mental health problems were evaluated using phi coefficients. To determine differences in maternal emotional availability for each EA scale (sensitivity, structuring, non-intrusiveness, non-hostility, child’s responsiveness, and child’s involvement) between the pre- and the post-lockdown groups simple t-tests have been computed in the next step. Lastly, the influence of maternal emotional availability in the pre- versus the post-lockdown group and the amount of infantile regulatory disorders was analyzed using bivariate correlations for the total score of each EAS scale and the main categories of the cumulative scores of infant regulatory disorders. Significance level for all analyses was set at α = 0.05. All data were analyzed using SPSS 29 [41].

## 3. Results

### 3.1. Descriptives

Table 1 shows the demographic characteristics of the study sample. Mothers were on average 34 years old, almost all of them were co-parents and living in a partnership, most of them had a high level of education and were in an economically sound situation. Results are presented depending on group membership (pre- vs. post-lockdown). The two groups of pre- and post-lockdown did not differ relevantly on any of the selected. For none of the demographic characteristics differences between pre- and post-lockdown group were revealed.

### 3.2. Infantile Regulatory Disorders

Regarding the comorbidities, the main category of cumulative sleeping disorders (having at least two diagnosed sleeping disorders) were found associated with the time of inclusion into the study (*ϕ* = 0.243; *p* = 0.016; Table 2). The explorative analyses of the subcategories of sleeping disorders revealed no further effect. No further infantile regulatory disorder was found to be associated with the pre- and the post-lockdown-group (all phi’s ≤ 0.122, *p*’s ≥ 0.222, Table 2).

### 3.3. Maternal Mental Health

**Figure 1:**
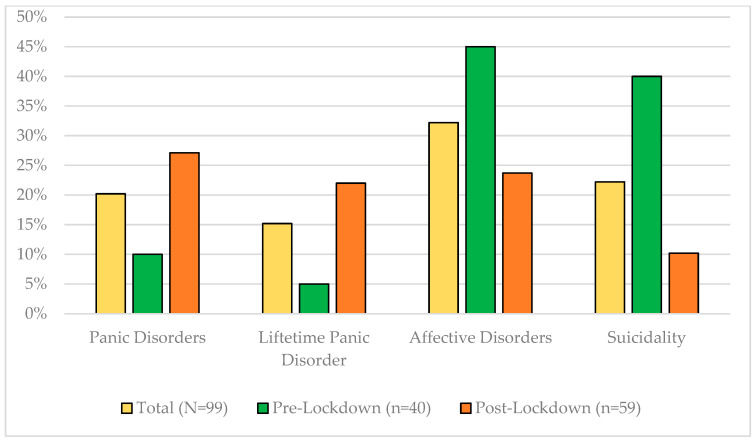
Maternal disorders with differences at *p* < 0.05 in the total sample and between the pre- and post-lockdown group.

Maternal panic and anxiety disorders, as well as affective disorders altered between participants included post-lockdown. Mothers examined in the pre-lockdown group had less often been diagnosed with a panic and anxiety disorder than mothers examined in the post-lockdown group. The explorative analyses revealed that only differences between groups for panic disorders (having at least one panic disorder) in general (*ϕ* = 0.229; *p* = 0.022) and lifetime panic disorders (*ϕ* = 0.231; *p* = 0.024) are visible. Furthermore, mothers examined after the onset of the first lockdown, less often had an affective disorder in general (*ϕ* = −0.355; *p* =< 0.001) and showed less suicidality (*ϕ* = −0.370; *p* =< 0.001) compared to the pre-lockdown group (Figure 1). All participants ranked as suicidal had the lowest suicidal ranking possible. All other differences concerning maternal disorders are not significant between the two groups.

### 3.4. Infantile Diagnoses and Maternal Mental Health

In the following, co-occurrences of infantile regulatory disorders and maternal disorders will be reported. Infantile sleeping disorders and maternal phobic disorders were associated in the post-lockdown group only (*ϕ* = 0.261; *p* = 0.045). Furthermore, infantile mood disorders and maternal affective disorders showed a strong association in the post-lockdown group (*ϕ* = 0.379; *p* = 0.004; Table 3).

### 3.5. Mother-Child-Interaction

**Figure 2:**
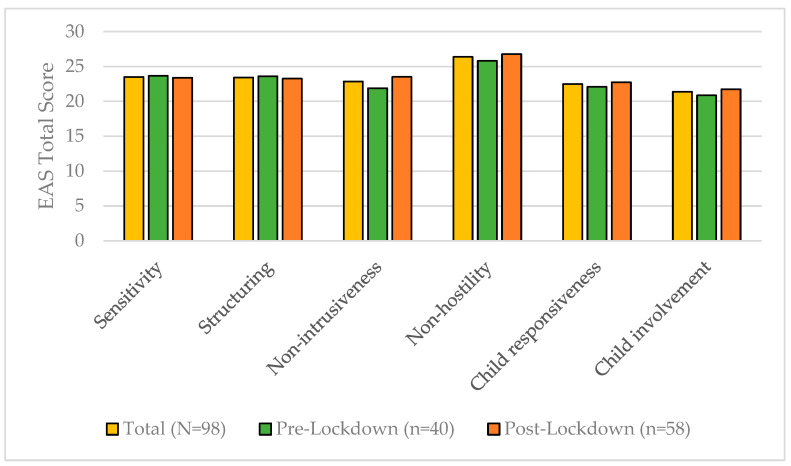
Emotional availability of mothers in the total sample and the pre- versus post-lockdown groups.

The total scores in our sample ranged from 10 to 29 (*M* = 23.329) on the six EA-scales (Figure 2). There was no relevant difference for any of the EA-scales between both groups, (all *t*’*s* ≤ 1.76, *p*’*s* > 0.081).

### 3.6. Mother-Child Interaction and Infantile Diagnoses

No differences at *p*< 0.05 could be observed for sensitivity, structuring, non-intrusiveness, non-hostility, child responsiveness, or child involvement pre- and post-lockdown for cumulative infantile disorders, but the explorative analysis shows that non-hostility might lose its promotive mechanism in pre- (*r* = −0.275; *p* = 0.037 *) versus post- (*r* = −0.069; *p* = 0.67) lockdown group. While there is a mid-to-strong association at *p* < 0.05 pre-lockdown, the direct comparison between both correlation coefficients is not (*z* = 1.002; *p* = 0.158).

## 4. Discussion

In the present study, we examined, based on a clinical sample of mothers and their children diagnosed with infantile regulatory disorders, if the frequency of infantile regulatory disorders and maternal mental health altered before and after the onset of the COVID-19 lockdown periods in Germany. We further examined if the mother-child interaction could be a contributing factor for the frequency of regulatory disorders pre- and post-lockdown.

Results show that maternal mental health and regulatory disorders of the child differ between pre- and post-lockdown. While studies pointed to the fact that maternal mental health problems could increase during the lockdowns [3], such findings could not be replicated in the present study. However, mothers who were recruited after the onset of the first lockdown showed significantly more panic and anxiety symptoms in general. These results are in line with previous findings [1,4,7,42], which evaluated changes in mental health during the pandemic and reported an increase of panic and anxiety symptoms in adults. It could be assumed that being socially isolated (such as having to stay at home or being afraid of infection) could have been a contributing factor for help-seeking behavior, see [43,44]. Mothers with panic or anxiety disorders may have sought less help before the lockdown, while this became more necessary during the lockdown periods. These findings do not support a general alteration in maternal mental health problems before and during the onset of the lockdown, but indicate a change in the study sample. In addition, while the frequency of lifetime panic disorders increased, affective disorders decreased. These findings seem to be at odds with previous studies predicting an overall increase of affective disorders during the COVID-19 pandemic [3,4,5]. On the other hand, the cumulation of stressors and isolation during the pandemic lockdown could result in staying at home or (at least) refusing study participation. The pandemic might present additional barriers like time constraints due to increased childcare responsibilities and can make it difficult for parents to seek help [43].

Infants and toddlers demonstrated significant differences in the occurrence of cumulative sleeping disorders after the onset of the first lockdown. Only one infant with cumulative sleeping disorders was included in the trial before the onset of the first German lockdown. After the beginning of the lockdowns, 16 infants and toddlers had more than one sleeping disorder simultaneously. The infant’s sleep was deeply affected by the day-to-day experiences, parental co-regulation, and possibly parental mental health. The pandemic lockdowns, as an exceptional situation, could have caused multiple stressors in the family system. According to Georg et al. [14], the impact of maternal psychological disorders can affect children’s regulatory functions. The social isolation and reduction of social peer interactions, make the children more vulnerable for maternal emotional availability alterations and ongoing disturbances in the interaction. The ongoing lockdowns and isolation appeared to cause more interactions at risk, resulting in the manifestation of regulatory disorders. An impeded affective communication (due to mask wearing or maternal psychopathology e.g., affective disorders) can further impede the children’s development. In addition, the impact of maternal psychological disorders on children’s regulatory functions is well known [6,7,14]. Maternal affective disorders showed the strongest association to infantile regulatory disorders. If the capacity of affect regulation for an infant is limited, the child is more likely to develop a regulatory disorder, often followed by later on behavioral problems [15,45,46]. In our sample, fewer maternal affective disorders were diagnosed in the post-lockdown group, but their impact might have remained more strongly on infantile mood disorders. Literature on this topic shows the impact of maternal (postpartum) depression and on co-regulation of the child and also mention the impact of affective disorders on children’s affect regulation capacity [45,47,48,49]. Furthermore, the social isolation and people wearing masks have an impact on the children’s social and emotional development in general [50], and could possibly have made the influence for vulnerable children more severe in the post-lockdown group. Aside from affective disorders, maternal phobic disorders showed an association to infantile sleep disorders in the post-lockdown group and support the assumed connection between maternal co-regulation capacities and infant’s sleep.

Lastly, we assessed to what extent the mother-child interaction amends during the pandemic and is itself associated with infantile regulatory disorders. There were no significant differences in emotional availability between the pre- and post-lockdown group. While most mentioned studies found alterations of emotional availability during the pandemic [28,29,31], Shakiba et al. [33] report that EA-scores are consistent before and during the pandemic in a study with infants. This is in line with our findings. Of interest is also that the emotional availability in the present sample is relatively high, thus, it is unlikely that floor effects reduced the possibility to find an association with lockdown phase. Before the onset of the first lockdown, maternal non-hostility seemed to reduce cumulative infantile psychopathologies. Less harsh parenting promotes psychological well-being of an infant or toddler [18,19,20,24]. This effect is not visible in mother-infant-dyads recruited after the onset of the first German lockdown—though the direct comparison of this correlation did not reveal any effect. Arguably, the new conditions evoked by the pandemic, with their rising levels of maternal fear and an unexplainable outside world, could have led to differences in the interaction [28,32]. While no differences in the levels of emotional availability were observed, the outcome might differ. It is fair to assume that infants and toddlers do not profit from positive mother-child interaction in the same way under such unknown pressure and therefore, the impact of promotive effects could decrease.

In the long run, these alterations might lead to less resilience towards cumulative infantile disorders in COVID-19 like situations. If resilience is understood as a state, rather than a personality trait, it represents a dynamic process and varies as a result of various adverse events [27,51,52]. According to this theory, the underlying mechanisms, such as promotive factors, might alter between different situations. The pandemic resulted in multiple stressors for mothers and their children: loss of resources (such as grandparents or nursery schools), economic problems, job loss, physical and mental health problems, and daily changing quarantine regulations. Coping with a completely new, adverse situation could affect the potential impact of promotive factors, such as non-hostility, and independence of the parenting function. In addition, a decrease in overall parental functioning during the pandemic has been described in other studies [32,53]. Therefore, further research is needed on the potential loss of effect for promotive factors in crisis situations, such as the COVID-19 outbreak, and the impact on infant and toddler resilience.

There are some limitations to the present study and its analyses. First, we did not differentiate between lockdown-weeks, quarantine, and local restrictions in the post-lockdown group. According to Brülhart et al. [54] the demand of professional help increased during the first lockdown week in Germany and slowly decreased after 3 weeks. The lack of findings could be related to this. Further, we did not assess any other parental report about (non)-parental care arrangements (like the amount of time spent with the infant), so we cannot differentiate how much the pandemic influenced the dyadic interaction. Secondly, regulatory symptoms were assessed in a self-constructed structured parental interview with the mother as key informant. Mothers who have a psychological disorder might evaluate their infants’ symptoms differently. Also, the pandemic may have caused more distress, which elevated the judgement of infantile symptom severity. Thirdly, we assessed a sample including only infants and toddlers with at least one regulatory disorder without any control group, i.e., a select clinical sample is described based on inclusion and active participation in a clinical trial. Fourthly, the sample size is limited, which leads to even smaller sample sizes per group and especially per disorder. Fifthly, we only examined data from participants either included before or after the onset of the first lockdown in Germany. There could be some overlapping effects for the structured interviews, while the assessment of EAS is a snapshot of the current situation. Few mothers were wearing masks during the recording of EAS videos, which made assessment very challenging. Lastly, it is possible that participants included right at the onset or after, have not been influenced strongly by the pandemic yet and vice versa. Although, the timeline in Figure S1 reveals that the first inclusion after the cut-off was approximately one month post lockdown.

## 5. Conclusions

Our main hypothesis was that infants and toddlers might develop psychological symptoms as a result of COVID-19 lockdown periods, although they were not directly affected by restraints. Although the data cannot confirm this hypothesis, they point to the fact, that there was a shift in maternal mental health problems (from affective to panic/anxiety disorders) and in children who were classified as having cumulative sleeping disorders. Infants and toddlers cannot be thought of without their parents due to their age. Arguably, the cumulation of stressors during the pandemic lockdown might have resulted in help-seeking behavior for the mothers with symptoms of panic and anxiety, while mothers with affective disorders might have stayed home or refused study participation. Maternal disturbances might have affected the child’s ability to co-regulate infantile affects post-lockdown, but not before. The present study hints in the direction of alterations in the promotive effects of emotional availability during the pandemic. Non-hostility loses its possible promotive mechanism towards infantile mental health. Although the results were not significant, the data points in the direction of differences in the regulatory competences of infants and toddlers in the COVID-19 pandemic. Assuming that the COVID-19 pandemic has an impact on maternal mental health and children’s regulatory disorders as well as their interaction behavior. The findings show that there is a need for further research to assess the impact of the pandemic on the child’s development and mental health of families. All in all, the pandemic situation allows us to assess the impact of the environmental and social experiences on infants’, toddlers’ and their parents’ development over time and future research should addressed to the long-term effects of the pandemic on the families.

All the above-mentioned results should be considered in treatment of young children and their families. Early regulatory disorders can very much influence the psychological well-being over time and a fluid transition in behavioral impairments is well described [15]. The treatment of the very young should be a societal focus. Treatment should implement the risks of the COVID-19 pandemic. Thus, in addition to the school-age children and adolescents who have already become the focus of care, attention should also be focused on infant and toddler mental health as a result of the COVID-19 pandemic.

## Figures and Tables

**Table 1:** Demographic characteristics of the study sample.

	Total (*N* = 99)	Pre-Lockdown (*n* = 40)	Post-Lockdown (*n* = 59)	Test Statistic	*p*
Mothers					
Mean Age (SD)	33.7 (4.9)	34 (4.9)	33.5 (4.9)	t_df(97)_ = 0.46	0.636
Age group (n, %)					
− ≤27	18 (18.2)	8 (20)	10 (16.9)		
− 28–39	71 (71.7)	28 (70)	43 (72.9)		
− 40–49	10 (10.1)	4 (10)	6 (10.2)		
Nationality (n, %)				Χ^2^_df(5)_ = 5.28	0.383
− German	63 (85.2)	29 (85.3)	34 (85)		
− Other country	11 (14.8)	5 (14.7)	6 (15)		
− Missing	25	6	19		
Custody of child				Χ^2^_df(2)_ = 1.62	0.445
− Shared	83 (90.2)	34 (87.2)	49 (92.5)		
− Only mother	6 (6.5)	4 (10.3)	2 (3.8)		
− Only father	0 (0)	0 (0)	0 (0)		
− Other	3 (3.3)	1 (2.6)	2 (3.8)		
− Missing	7	1	6		
Educational level				Χ^2^_df(4)_ = 5.32	0.256
− Low	5 (6.8)	1 (2.9)	4 (10.3)		
− Middle	16 (21.9)	10 (29.4)	6 (25.6)		
− High	52 (71.2)	23 (67.7)	29 (74.4)		
− Missing	26	6	20		
Relationship status				Χ^2^_df(1)_ = 0.65	0.421
− Currently in a partnership	65 (92.9)	27 (90)	38 (95)		
− Single parent	5 (7.1)	3 (10)	2 (5)		
− Missing	29	10	19		
Number of children in the household				Χ^2^_df(2)_ = 1.63	0.443
− 1	34 (54)	14 (46.7)	20 (60.6)		
− 2	23 (36.5)	12 (40)	11 (33.3)		
− 3 or more	6 (9.5)	4 (13.3)	2 (6.1)		
− Missing	36	10	26		
Financial situation				Χ^2^_df(2)_ = 2.29	0.319
− Sufficient	61 (88.4)	31 (91.2)	30 (85.7)		
− Low	7 (10.1)	2 (5.9)	5 (14.3)		
− Low + in debt	1 (1.4)	1 (2.9)	0		
− Missing	30	6	24		
Children					
Mean Age in months (SD)	18.7 (10.8)	19.8 (11.2)	17.9 (10.6)	t_df(97)_ = 0.88	0.383
Age group (n, %)					
− 0–12 months	33 (33.3)	13 (32.5)	20 (33.9)		
− 13–24 months	30 (30.3)	10 (25)	20 (33.9)		
− 24–36 months	36 (36.4)	17 (42.5)	19 (32.2)		
Gender (n, %)				Χ^2^_df(1)_ = 1.50	0.220
− Female	47 (47.5)	16 (40)	31 (52.5)		
− Male	52 (52.5)	24 (60)	28 (47.5)		

**Table 2:** Infantile regulatory disorders in the pre- and post-lockdown group plus comparison of cumulative infantile regulatory disorders in the pre- and post-lockdown group derived from structured interview.

	Pre-Lockdown	Post-Lockdown	Test Statistics	
	(*n*; %)	(*n*; %)	*ϕ*	*p*
Sleeping Disorders	13 (32.5)	11 (42.4)	0.100	0.322
Cumulative Sleeping Disorders	1 (2.5)	11 (18.6)	0.243	0.016 *
Eating Disorders	18 (45.0)	26 (44.1)	0.010	0.927
Cumulative Eating Disorders	4 (10)	5 (8.5)	0.026	0.796
Mood Disorders	6 (15.0)	9 (15.3)	0.003	0.972
Cumulative Mood Disorders	1 (2.5)	0 (0)	0.122	0.222
Other Disorders	16 (40.0)	19 (32.2)	0.080	0.426
Cumulative Other Disorders	3 (7.5)	2 (3.4)	0.092	0.359
Any Regulatory Disorder	32 (80.0)	46 (78.0)	0.060	0.808
Cumulative Regulatory Disorders	19 (47.5)	31 (52.5)	0.242	0.622

Note. Shown are the cumulative scores of the four main categories of infantile regulatory disorders; *ϕ* = phi coefficient; * *p* < 0.05.

**Table 3:** Associations between infantile regulatory disorders and maternal disorders in the pre- versus post-lockdown group.

	Pre- Lockdown	Test Statistics		Post- Lockdown	Test Statistics	
	(*n*; %)	*ϕ*	*p*	(*n*; %)	*ϕ*	*p*
Infantile Sleeping Disorders						
Maternal Panic and Anxiety	2 (5)	0.102	0.519	4 (6.8)	−0.193	0.138
Maternal Affective Disorders	7 (17.5)	0.124	0.433	7 (11.9)	−0.015	0.911
Maternal Phobic Disorders	1 (2.5)	0.005	0.974	6 (10.2)	0.262	0.045 *
Maternal Eating Disorders	0 (0)	−0.111	0.482	1 (1.7)	0.153	0.240
Infantile Eating Disorders						
Maternal Panic and Anxiety	1 (2.5)	−0.152	0.336	9 (15.3)	0.061	0.642
Maternal Affective Disorders	11 (27.5)	0.234	0.140	9 (15.3)	0.098	0.453
Maternal Phobic Disorders	1 (2.5)	−0.067	0.673	5 (8.5)	0.147	0.259
Maternal Eating Disorders	1 (2.5)	0.177	0.263	0 (0)	−0.117	0.371
Infantile Mood Disorders						
Maternal Panic and Anxiety	2 (5)	0.175	0.268	5 (8.5)	0.241	0.064
Maternal Affective Disorders	2 (5)	−0.036	0.819	7 (11.9)	0.379	0.004 *
Maternal Phobic Disorders	1 (2.5)	0.146	0.355	3 (5.1)	0.245	0.060
Maternal Eating Disorders	0 (0)	−0.067	0.671	0 (0)	−0.056	0.669
Infantile Cumulative All Disorders						
Maternal Panic and Anxiety	1 (2.5)	0.066	0.667	2 (3.4)	−0.078	0.548
Maternal Affective Disorders	0 (0)	0.129	0.414	2 (3.4)	0.015	0.909
Maternal Phobic Disorders	0 (0)	−0.095	0.548	2 (3.4)	0.211	0.106
Maternal Eating Disorders	0 (0)	0.080	0.613	0 (0)	0.070	0.592

Note. *ϕ* = phi coefficient; * *p* < 0.05.

## Data Availability

The data presented in this study are available on reasonable request from the corresponding author. The data are not publicly available due to the private details of the participants they contain.

## Supplementary Materials

The following supporting information can be downloaded at: https://www.mdpi.com/article/10.3390/children10121885/s1. Figure S1. Participant’s date of inclusion (dd/mm/yy); Green representing inclusion before the onset of the first German lockdown, orange the inclusion afterwards. Table S1. Infantile regulatory disorders according to the DC: 0–5TM. Only considered disorders of infantile regulation are listed below. Table S2. Psychiatric disorders by DSM IV and ICD-10 evaluated by the M.I.N.I. psychiatric interview.
