# Supplementary material for: Being an Infant in a Pandemic: Influences of the COVID-19 Pandemic on Infants, Toddlers and Their Mothers in a Clinical Population
Source: Children (Basel). 2023 Dec 1;10(12):1885. doi: 10.3390/children10121885 (PMC10742006; doi:10.3390/children10121885)
Supplement: Supplementary file 1 [file children-10-01885-s001.zip › children-2720914-supplementary.pdf]

# Supplementary Materials

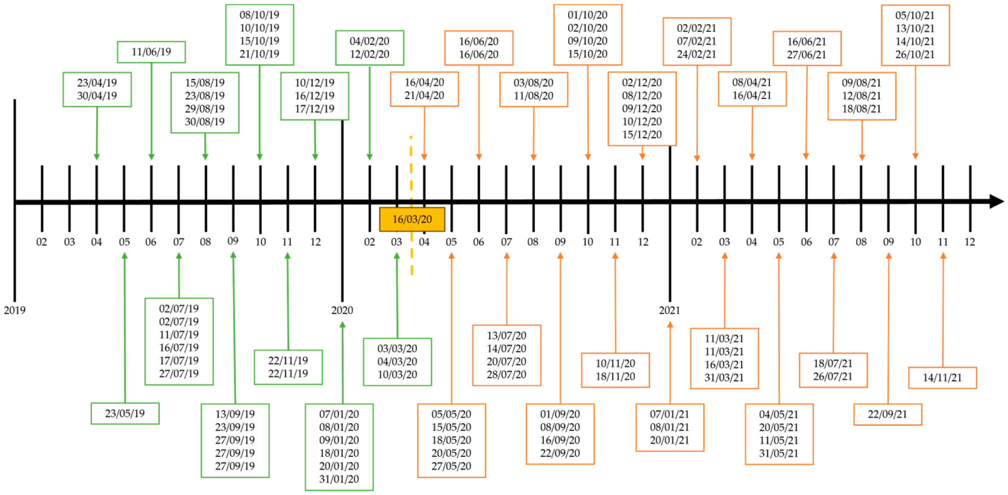


**Figure S1.** Participant's date of inclusion (dd/mm/yy); Green representing inclusion before the onset of the first German lockdown, orange the inclusion afterwards.

**Table S1** Infantile regulatory disorders according to the DC: 0–5™. Only considered disorders of infantile regulation are listed below.

| **Sleeping, Eating and Crying Disorders** |
|---|
| Sleep Onset |
| Night Waking |
| Partial Arousal |
| Nightmares |
| Overeating |
| Undereating |
| Atypical Eating |
| Excessive Crying |
| Other Sleep, Eating, and Excessive Crying Disorder of Infancy or Early Childhood |
| **Mood Disorders** |
| Depression |
| Dysregulated Anger and Aggression |
| Other Mood Disorder |
| **Anxiety Disorders** |
| Separation Anxiety |

**Table S2.** Psychiatric disorders by DSM IV and ICD-10 evaluated by the M.I.N.I. psychiatric interview.

| |
|---|
| Major depressive episode: current |
| Major depressive episode: lifetime |
| Major depressive episode with melancholia |
| Dysthymia |
| Suicidality |
| (Hypo-)manic episode: current |
| (Hypo-)manic episode: lifetime |
| Panic disorder: current |
| Panic disorder: lifetime |
| Panic disorder with few symptoms |

| |
|---|
| Panic disorder without agoraphobia |
| Panic disorder with agoraphobia |
| Agoraphobia without former panic disorder |
| Social phobia |
| Obsessive-compulsive disorder |
| Posttraumatic stress disorder |
| Alcohol addiction |
| Alcohol abuse |
| Drug addiction |
| Drug abuse |
| Psychotic disorders |
| Anorexia nervosa |
| Anorexia nervosa: Binge-Eating |
| Bulimia nervosa |
| Generalized anxiety disorder |
| Antisocial personality disorder |
